# The *hexokinase* Gene Family in Cotton: Genome-Wide Characterization and Bioinformatics Analysis

**DOI:** 10.3389/fpls.2022.882587

**Published:** 2022-05-16

**Authors:** Lingling Dou, Zihan Li, Huiqin Wang, HuaiZhu Li, Guanghui Xiao, Xianliang Zhang

**Affiliations:** ^1^School of Chemistry and Chemical Engineering, Xianyang Normal University, Xianyang, China; ^2^State Key Laboratory of Cotton Biology, Institute of Cotton Research, Chinese Academy of Agricultural Sciences, Anyang, China; ^3^College of Life Sciences, Shaanxi Normal University, Xi’an, China

**Keywords:** *Gossypium hirsutum*, hexokinase, sequence analysis, the evolutionary, expression pattern

## Abstract

Hexokinase (HXK) is involved in hexose phosphorylation, sugar sensing, and signal transduction, all of which regulate plant growth and adaptation to stresses. *Gossypium hirsutum* L. is one of the most important fiber crops in the world, however, little is known about the *HXKs* gene family in *G. hirsutum* L. We identified 17 *GhHXKs* from the allotetraploid *G. hirsutum* L. genome (AADD). *G. raimondii* (DD) and *G. arboreum* (AA) are the diploid progenitors of *G. hirsutum* L. and contributed equally to the At_genome and Dt_genome *GhHXKs* genes. The chromosomal locations and exon-intron structures of *GhHXK* genes among cotton species are conservative. Phylogenetic analysis grouped the HXK proteins into four and three groups based on whether they were monocotyledons and dicotyledons, respectively. Duplication event analysis demonstrated that HXKs in *G. hirsutum* L. primarily originated from segmental duplication, which prior to diploid hybridization. Experiments of qRT-PCR, transcriptome and promoter *cis*-elements demonstrated that *GhHXKs’* promoters have auxin and GA responsive elements that are highly expressed in the fiber initiation and elongation stages, while the promoters contain ABA-, MeJA-, and SA-responsive elements that are highly expressed during the synthesis of the secondary cell wall. We performed a comprehensive analysis of the *GhHXK* gene family is a vital fiber crop, which lays the foundation for future studies assessing its role in fiber development.

## Introduction

Carbohydrates produced by photosynthesis are eventually stored as sugar. Sugar has an important influence on various stages of the plant life cycle, and can be converted to fructose and glucose in the reservoir tissue (Desnoues et al., 2014). Fructose and glucose are essential six-carbon sugars in plants, which are also known as hexose. Hexose can be phosphorylated by the enzyme of hexokinases (HXKs) (Jang et al., 1997; Halford et al., 1999). Phosphorylated hexose has diverse functions, including the following: phosphorylated hexose is in an activated form that readily participates in metabolic reactions; phosphorylated glucose molecules have a robust polar group and can effectively prevent intracellular hexose extravasation; phosphorylated glucose can store the phosphate group, which could be converted to the terminal high-energy phosphate group of adenosine diphosphate (ADP) (Etienne et al., 2002; Desnoues et al., 2014). Therefore, typical HXKs contain glucose binding domain and adenosine phosphate binding domain (Karve et al., 2008). HXKs play diverse roles in regulating plant growth and function as sugar sensors, regulate sugar signal transduction, and cooperate with phytohormones.

Recent studies have demonstrated that hexokinase is involved in sugar sensing and signal transduction in plants, while AtHXK1 functions as a glucose sensor. The plants of 35S:sense-*AtHXK1* is hypersensitive to glucose with small cotyledons, hypocotyls, and roots; 35S-antisense-*AtHXK1* transgene plants are hypersensitive to glucose and are typically grown with green, expanded cotyledons and root elongation under glucose treatment (Jang et al., 1997). The mutant *ScHXK1* showed that hexokinase had a glucose-sensing function, independent of its enzymic activity in *Saccharomyces cerevisiae* (Mayordomo and Sanz, 2001).

Sugar functions as a potential signaling molecules throughout a plant’s life cycle (Hanson and Smeekens, 2009), which is independent of its enzymatic role in converting glucose to glucose 6-phosphate (Smeekens et al., 2010). AtHXK1 is involved in programmed cell death in *Arabidopsis*, which is mediated by *myo*-*inositol* accumulation (Bruggeman et al., 2015). Sugar induced during cell death depends on the rate of AtHXKs-induced sugar phosphorylation; while in yeast, the affinity of AtHXK is higher for glucose than for fructose (Granot and Dai, 1997).

Sugars are a signal in regulating plant growth and cooperate with phytohormones (Smeekens, 2000). The autophagy regulating AtHXK1-dependent glucose signaling-mediated root meristem activity functions by modulating the production of reactive oxygen species (ROS) in *Arabidopsis* (Huang et al., 2019). ABA application can improve the expression of *sucrose synthases* (*SuSys*) and *cell wall invertase* (*CWINV*), and block the glucose-induced repression of two genes, which are insensitive to glucose treatment in *CsHXK1* or *CsHXK2* mutants (Wang et al., 2017).

In tomato plants, the *SlHXK1* mutant showed enhanced leaf senescence and repressed plant growth by affecting starch turnover (Li et al., 2020). *OsHXK1*-CRISPR/Cas9 plants showed increased plant light tolerance, photosynthetic products, and rice yields along with a significantly increased expression of photosynthesis-related genes (Zheng et al., 2021). The exogenous application of glucose to *Arabidopsis* can promote true leaf expansion in an *AtHXK1*-dependent manner; however, the increased expression of *AtHXK1* inhibited leaf expansion (Xiao et al., 2000). The upregulation of *OsHXK1* increased glucose and ROS levels and promoted programmed cell death (PCD) and leaf senescence (Zheng et al., 2021). HXKs are involved in the steady-state recycling of ADP, while ADP content also regulates H_2_O_2_ formation on the mitochondrial inner membrane (Valluru and Van den Ende, 2011).

As sequencing technology develops, the economic value of cotton fibers increases and the genomes of *Gossypium hirsutum* L. (AADD, 2n = 4X = 52) (Paterson et al., 2012; Li et al., 2015; Hu et al., 2019; Wang et al., 2019), *G. anomalum* (BB, 2n = 2X = 26) (Grover et al., 2021a), *G. stocksii* (EE, 2n = 2X = 26) (Grover et al., 2021b), *G. longicalyx* (FF, 2*n* = 2*X* = 26) (Grover et al., 2020), and *G. rotundifolium* (KK, 2n = 2X = 26) (Wang et al., 2021), etc. have all been sequenced.

*Gossypium hirsutum* L. is the most widely spread cotton species; it accounts for 90% of all cotton species produced in the world (Li et al., 2015). *G. hirsutum* L. fibers are highly specialized epidermal hair cells formed on the surface of a seed. They have a single cell structure formed by the protuberance, differentiation and elongation of epidermal cells inside and outside the ovary of the ovule. Cotton fiber differentiation and development can be divided into four stages: the fiber initiation stage, which occurs 3 days before flowering to 3 days post-anthesis (−3 to 3 DPA); the rapid elongation stage, which occurs in the fiber cells from 5 to 25 DPA (Qin and Zhu, 2011); the thickening stage of the cell wall (20–45 DPA); and the fiber dehydration and maturation stage (45–50 days) (Wu et al., 2017).

Studies assessing the development of cotton fibers have demonstrated that hexokinase is involved in glucose-mediated fiber elongation, that low glucose levels promoted cotton fiber elongation, and that treatment with hexokinase inhibitor N-acetyl-glucosamine (NAG) inhibited fiber elongation (Li et al., 2021). Considering the essential functions of *GhHXKs* in sugar conversion and signal transduction during fiber elongation process in cotton, we performed a genome-wide analysis of *GhHXKs* and characterized the structure and expression patterns of *GhHXKs*.

## Materials and Methods

### Plant Growth and Treatment

A *G. hirsutum* cultivar, Xuzhou 142, was planted in the greenhouse with a 16 h light, 30°C/8 h dark, 30°C cycle, as previously reported (He et al., 2017). For phytohormone treatment, 0 DPA fresh ovules were collected from cotton bolls, sterilized, and cultured in previously reported liquid culture medium (Shi et al., 2006), which added with 5 μM 1-Naphthylacetic acid (NAA, Sigma) and 1 μM gibberellin acid (GA3, Sigma) for the indicated time (He et al., 2019), respectively. After treatment, the ovules were collected for quantitative real-time (qRT-PCR) experiments. For RNA extraction, fresh cotton seed fibers were harvested from 0, 5, 10, 15, 20, and 25 DPA, and then immediately frozen in liquid nitrogen.

### Molecular Databases

The genome sequences of *G. hirsutum* L. genome (NDM8), *G. raimondii* (JGI_v2.1), *G. arboreum* (CRI_v3.0), *G. anomalum* (NSF_v1), *G. stocksii* (NSF_v1), *G. longicalyx* (NSF_v1), and *G. rotundifolium* (HAU v1) were downloaded from CottonGen^1^ (Ma et al., 2021). The genome sequence of *Arabidopsis thaliana* was downloaded from The Arabidopsis Information Resource (TAIR^2^) database (Lamesch et al., 2012). The *HXK* sequences from *O. sativa* (Cho et al., 2006), *Phyllostachys edulis* (Moso Bamboo) (Zheng et al., 2020), and *Manihot esculenta* (Cassava) (Geng et al., 2017) were downloaded from the Nucleotide database.^3^ The genome sequence (Chalhoub et al., 2014) of *Brassica napus* was downloaded from the Brassicaceae Database (BRAD^4^). The genome size, sequences and taxonomy ID of *Ostreococcus lucimarinus*, *Chlamydomonas reinhardtii*, Volvox carteri, *Coccomyxa subellipsoidea*, *Chlorella variabilis*, and *Selaginella moellendorffii* were downloaded from the Genome database of NCBI.^5^

### Identification of Hexokinase Members

Two HXK Pfam domains (PF03727 and PF00349) were used to search against the *G. hirsutum* L., *G. raimondii*, *G. arboreum*, *G. anomalum*, *G. stocksii*, *G. longicalyx*, and *G. rotundifolium* genomes using the hidden Markov model (HMM) with HMMER 3.0 (Prakash et al., 2017). The candidate GhHXKs, GaHXKs, GrHXKs, GanHXKs, GstHXKs, GloHXKs, and GroHXKs were submitted to the SMART software (Letunic et al., 2021)^6^ and the Conserved Domain Database (Lu et al., 2020) (CDD^7^) to confirm that all candidate HXK proteins contained the Hexokinase domain.

We used the general feature format (GFF) file of the genomes to determine the relative position of *HXKs* on chromosomes, and visualized the locations with the online software MG2C (Jiangtao et al., 2015). Furthermore, the gene structures of *HXKs* were also analyzed according to the GFF files, and the “exon-intron” structure was shown by the Gene Structure Display Server (Hu et al., 2015) (GSDS 2.0^8^).

### Sequence Analysis

Protein motif analysis was performed using MEME^9^ with a maximum of eight motifs and using other default parameters.

The physicochemical properties, including molecular weight (MW), isoelectric point (pI), instability index, and grand average of hydropathicity (GRAVY), were analyzed using the online software ExPASy ProtParam tool (Artimo et al., 2012)^10^ in *GhHXKs*, *GaHXKs*, *GrHXKs*, *GanHXKs*, *GstHXKs*, *GloHXKs*, and *GroHXKs*, respectively.

The subcellular localization of the candidate HXKs were predicted by the online software, WoLF PSORT (Horton et al., 2007).^11^

### Phylogenetic Tree Construction

The HXK protein sequences of *G. hirsutum* L., *G. raimondii*, *G. arboreum*, *A. thaliana*, *O. sativa, P. edulis*, *M. esculenta*, and *B. napus* were aligned using ClustalW, and the evolutionary tree was constructed using the neighbor-joining method with MEAG 7.0 (Kumar et al., 2016). To evaluate the reliability of the phylogenetic tree, the bootstrap value was set as 1,000.

### Evolutionary Analysis

The duplication types of *GhHXKs*, *GaHXKs*, and *GrHXKs* were analyzed using the Multiple Collinearity Scan (MCScanX) toolkit under the Linux system (Yupeng et al., 2012). The orthologous- and homologous-gene pairs were visualized by the CIRCOS software (Krzywinski et al., 2009). The synonymous substitution rate (Ks), non-synonymous substitution rate (Ka), and Ka/Ks ratios were calculated using the KaKs_Calculator software (Wang et al., 2010). The divergence time between the homologous- and orthologous–gene pairs was calculated according to previously used methods (Yang et al., 2006).

### *Cis*-Acting Element Analysis of Promoter

The sequence 2,000 bp upstream of the initiation codon was extracted as the candidate promoters with the “fastacmd –d database –s chromosome –L start location, end location –o result” using the local BLAST software (Camacho et al., 2009). The *Cis*-elements in the candidate promoter sequence were analyzed by Plant *Cis*-acting Regulatory Element (Plant CARE^12^) (Lescot et al., 2002).

### Spatial and Temporal Expression Analysis of *GhHXK* Genes

The allotetraploid cotton cultivar, Xuzhou 142, was grown in Shaanxi Normal University under controlled conditions (He et al., 2017). A total of 30 ovules were used for each phytohormone and were performed in triplicate. Cotton ovules were collected at one DPA, sterilized with sodium hypochlorite (NaClO, 10%), and cultivated as previously reported (Shi et al., 2006). Five μM 1-Naphthylacetic acid (NAA, Sigma, Germany) and 1 μM GA_3_ (Sigma, Germany) were added to the culture medium. The ovules treated with phytohormones were used to perform RNA-seq, while the data was conserved in our lab (He et al., 2019).

To illustrate the spatial and temporal expression patterns of *GhHXKs*, the transcriptomes of various tissues (stamen, anther, seed, fiber, ovule, petal, calycle, torus, leaf, stem, root, cotyledon, stigma, and pistil) and a successive fiber developmental stages (0, 5, 10, 15, 20, 25, 30, and 35 DPA) were downloaded from NCBI (accession NO. PRJNA680449) (Ma et al., 2021). The expression data were normalized and visualized using Omicshare tools.^13^

### RNA Extraction and qRT-PCR Analysis

The total RNA extraction was performed according the instructions for the RNAprep Pure Plant Plus Kit (Code No. DP441, TIANGEN, China), and the cDNA was reverse-transcribed from 2 μg total RNA (Xiao et al., 2016). The qRT-PCR was conducted with three biological and three technical replicates as the following reaction parameters: 95°C for 30 s, followed by 40 cycles of 95°C for 5 s, 60°C for 15s, and 72°C for 20 s. A melting curve was generated from 65 to 95°C. The ubiquitin gene *GhUBQ7* (GenBank accession no. AY189972) was used as the internal control for each qPCR experiment. Primers for qRT-PCR experiments were listed in Supplementary Table 1.

## Results

### Identification and Characterization of *hexokinase* Genes From Cotton Species

To identify *HXK* genes in cotton species, two hexokinase domains (PF03727 and PF00349) were used as the query domains with the HMMER 3.0 software (on a Windows system) to search against the genomes of *G. hirsutum* L. (NDM8), *G. arboreum* (CRI_v3.0), *G. raimondii* (JGI_v2.1), *G. anomalum* (NSF_v1), *G. stocksii* (NSF_v1), *G. longicalyx* (NSF_v1), and *G. rotundifolium* (HAU_v1). There were 17 GhHXKs, 9 GaHXKs, 8 *GrHXKs*, 8 GanHXKs, 8 GstHXKs, 7 GloHXKs, and 8 GroHXKs retrieved from *G. hirsutum* L., *G. arboreum*, *G. raimondii*, *G. anomalum*, *G. stocksii*, *G. longicalyx*, and *G. rotundifolium*, respectively (Table 1 and Supplementary Table 2).

**TABLE 1:** Detailed information about HXKs in *G. hirsutum* L. genome.

Gene ID	Gene name	Chromosome location	Strand	Number of amino acids	Molecular weight (kDa)	Theoretical pI	Instability index	Aliphatic index	Grand average of hydropathicity (GRAVY)
GhM_A05G1470.1	GhHXK1	A05:12791661–12797197	–	415	45.59	5.97	34.98	90.46	–0.009
GhM_A06G0048.1	GhHXK2	A06:325131–327819	–	495	53.08	5.27	40.67	90.85	0.003
GhM_A06G0798.1	GhHXK3	A06:16658340–16667651	–	498	53.58	6.1	32.99	94.04	–0.018
GhM_A09G1097.1	GhHXK4	A09:60270679–60276064	+	496	54.02	5.96	33.34	93.57	–0.066
GhM_A10G0721.1	GhHXK5	A10:8080756–8083803	+	497	54.20	6.85	31.48	94.73	–0.015
GhM_A11G0599.1	GhHXK6	A11:4995581–4998261	–	492	53.85	6.87	33.67	88.21	–0.153
GhM_A13G2576.1	GhHXK7	A13:107006991–107011839	–	498	54.08	6.04	28.38	95.38	–0.033
GhM_A13G2808.1	GhHXK8	A13:109799752–109804195	+	504	54.82	7.1	46.27	98.99	0.034
GhM_D05G1484.1	GhHXK9	D05:11463058–11466549	–	386	41.87	5.96	35.31	91.74	0.011
GhM_D06G0045.1	GhHXK10	D06:272501–275218	–	495	53.09	5.8	37.83	88.48	–0.026
GhM_D06G0803.1	GhHXK11	D06:12259048–12269356	–	371	40.65	5.97	38.44	90.4	–0.073
GhM_D09G1032.1	GhHXK12	D09:36391164–36396448	+	496	54.02	6.21	32.04	93.77	–0.054
GhM_D10G0700.1	GhHXK13	D10:7279892–7282506	+	384	41.92	7.17	32.93	94.66	0.009
GhM_D10G0701.1	GhHXK14	D10:7282522–7287260	+	137	15.07	6.74	27.6	90.44	–0.292
GhM_D11G0594.1	GhHXK15	D11:4645981–4648915	–	492	53.79	7.18	35.58	90	–0.127
GhM_D13G2490.1	GhHXK16	D13:61037992–61042807	–	498	53.97	5.84	30.85	93.25	–0.043
GhM_D13G2694.1	GhHXK17	D13:63381196–63385591	+	504	54.94	6.49	46.32	99.17	0.021

The amino acids ranged from 137 (GhHXK14) to 504 (GhHXK8 and GhHXK17). The molecular weight of GhHXKs ranged from 15.07 kDa (GhHXK14) to 54.94 kDa (GhHXK17). According to isoelectric point (pI) analysis, 14 GhHXKs had pI less than 7.0 (with an average of 6.15) and were acidic proteins. In contrast, three GhHXKs were predicted to encode proteins more than 7.0 (average of 7.15) and were basic. Grand average of hydropathicity (GRAVY) analysis found that 12 GhHXKs with GRAVY scores less than zero were hydrophilic proteins; and that five GhHXKs with GRAVY scores more than zero were hydrophobic proteins. Based on the instability index analysis, 14 GhHXK proteins have instability index values less than 40.0 and three GhHXK proteins have instability index values greater than 40.0 (GhHXK2, GhHXK8, and GhHXK17). The detailed physicochemical properties of *GaHXKs*, *GrHXKs*, *GanHXKs*, *GstHXKs*, *GloHXKs*, and *GroHXKs* are listed in Supplementary Table 2.

### Chromosomal Location Analysis of *HXKs* in Cotton Species

**FIGURE 1:**
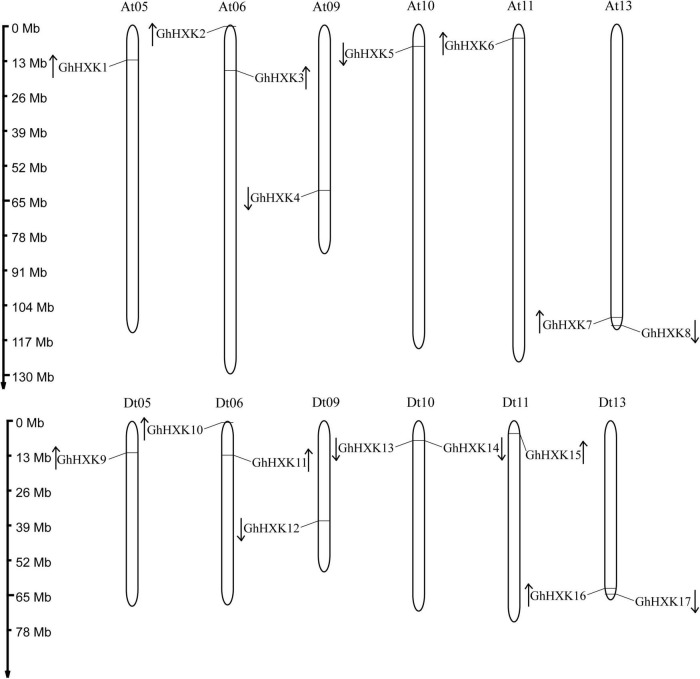
Chromosomal distribution of *GhHXKs*. The chromosome number is shown above each chromosome. The chromosomal location of each *GhHXKs* is shown from the top to the bottom of the corresponding chromosome. The scale bars beside the chromosome indicate the length of megabases (Mb). The arrows show the transcription directions of *GhHXK* genes.

**FIGURE 2:**
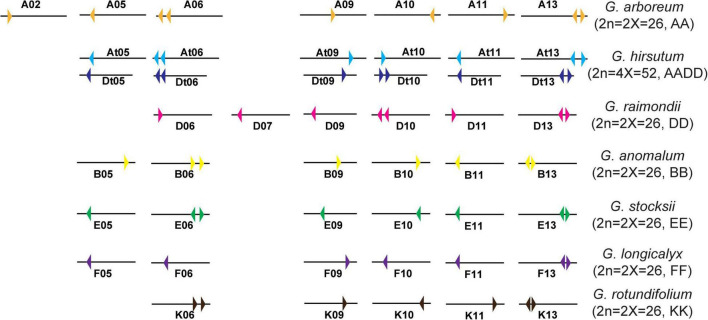
Chromosome distribution diagram of *HXK* genes in *G. hirsutum* L., *G. arboreum*, and *G. raimondii*. Colorful triangles represent the HXK genes and their transcription direction.

According to the GFF files of *G. hirsutum* L. (NDM8), the 17 *GhHXKs* are distributed on 12 *G. hirsutum* L. chromosomes. The 17 *GhHXKs* genes were named *GhHXK1* to *GhHXK17* from chromosomes At01 to Dt13 based on their relative chromosomal locations from the chromosome top to bottom (Figures 1, 2). There are eight *GhHXKs* distributed on six At_subgenomes (At05, At06, At09, At10, At11, and At13) and nine *GhHXKs* distributed on six Dt_subgenomes (Dt05, Dt06, Dt09, Dt10, Dt11, and Dt13). The *GhHXK* genes are evenly distributed on At_ and Dt_subgenomes, except for *GhHXK14*. Nine *GaHXKs* are distributed on seven *G. arboreum* genomes. The distribution of *GaHXK* genes across chromosomes was similar to that of *GhHXKs* on At_subgenome in *G. hirsutum* L., while there was an extra on Ga_Chr02 of *G. arboreum*. Eight *GrHXKs* were distributed on six chromosomes, which was similar to the distribution of *GhHXKs* on the Dt_subgenome in *G. hirsutum* L. At the same time, there is one more gene distributed on the chromosome Gr_Chr07 and one lost gene on the chromosome Gr_Chr05 in *G. raimondii* (Supplementary Figure 1). This indicated that their gene loss or duplicated evince existed in the *G. hirsutum* L. genome.

Furthermore, combined with GFF annotation files for other cotton species, the eight *GanHXKs* were distributed on six *G. anomalum* (2n = 2X = 26, BB) chromosomes, including B05, B06, B09, B10, B11, and B13, while there were eight *GstHXKs* distributed on six *G. stocksii* (2n = 2X = 26, EE) chromosomes, including E05, E06, E09, E10, E11, and E13. The seven *GloHXKs* were distributed on the identical chromosomes of *G. longicalyx* (2n = 2X = 26, FF) and were also distributed across the identical chromosomes of *G. rotundifolium* (2n = 2X = 26, KK), except for K05 (Figure 2). The distribution analysis demonstrated that the *HXKs* were conservatively distributed on the 5th, 6th, 9th, 10th, 11th, and 13th chromosomes among cotton species. According to the detailed distribution and transcription direction of *HXKs* between cotton species (Figure 2 and Supplementary Figures 1, 2), inversion and segmental duplication existed in the chromosomes of these cotton species’.

### Gene Phylogenetic and Structure of *GhHXKs*

**FIGURE 3:**
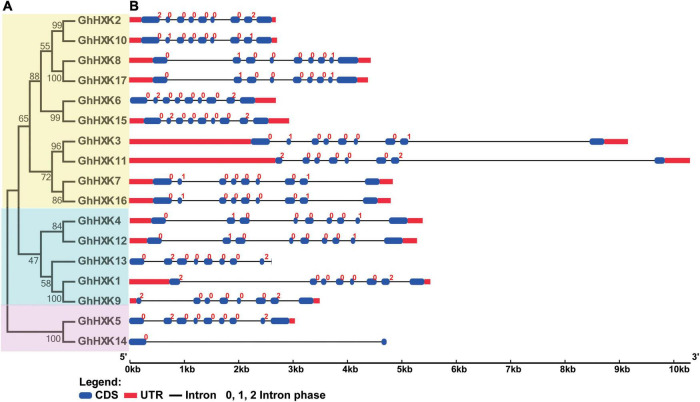
The phylogenetic tree **(A)** and gene structure **(B)** of *GhHXKs*. The phylogenetic tree was constructed with the CDS sequences of *GhHXKs* by MEGA 7.0 software with default parameters. The exons, UTR, and introns are indicated by blue ovals, red rectangles, and black lines, respectively.

In general, nucleic acid sequences are more variable than protein sequences. To well illustrate the evolutionary relationships among *GhHXKs*, a CDS phylogenetic tree was constructed by MEGA 7.0 (Figure 3A). According to the phylogenetic tree in Figure 3A, the *GhHXK* genes could be clustered into three groups.

The gene structures of *GhHXKs* were determined by assessing the annotation information of the GFF files in the *G. hirsutum* L. genome (NDM8), which were visualized using the GSDS 2.0 online software. The results demonstrated that most *GhHXKs* contained nine exons and eight introns, and four *GhHXKs* contained eight exons and seven introns, including *GhHXK1*, *GhHXK9*, *GhHXK11*, and *GhHXK13*. *GhHXK14* contained two exons and one intron. The 13 *GhHXKs* genes include both 5′- and 3′-UTRs, two *GhHXKs* contain 3′-UTR, while the remaining two genes (*GhHXK13* and *GhHXK14*) have no UTR region (Figure 3B). Studies assessing the gene structure of other cotton species (Supplementary Figure 3B) demonstrated that most *HXKs* in cotton species are conservative and have “intron-exon” structures.

### Phylogenetic Analysis of GhHXK Proteins

To illustrate the phylogenetic relationships between the HXKs proteins in *G. hirsutum* L. and those of other species, including *G. arboreum*, *G. raimondii*, *O. sativ*a, *A. thaliana*, *P. edulis*, *M. esculenta*, and *B. napu*s, an unrooted neighbor-joining tree was created using the MEGA 7.0 software based on their entire length of the amino acid sequences.

**FIGURE 4:**
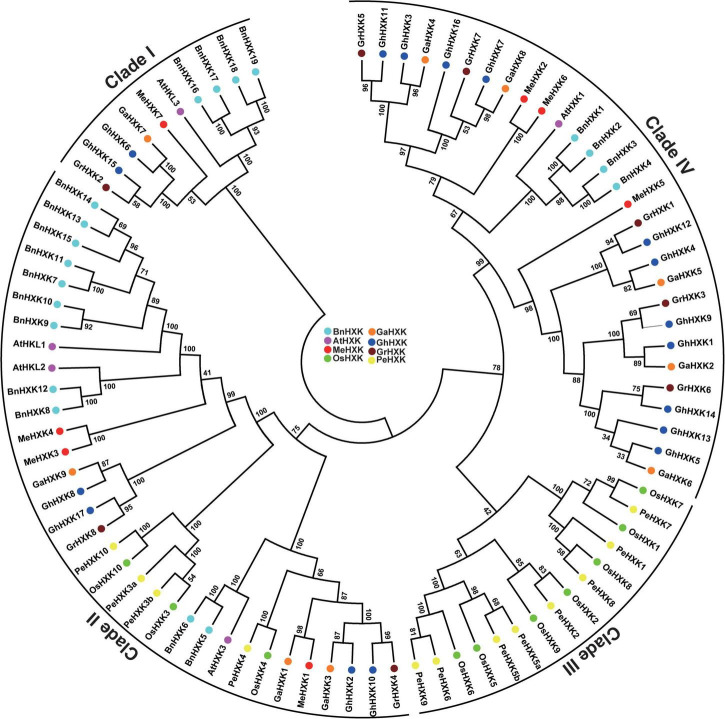
Phylogenetic analysis of GhHXK proteins in *G. hirsutum* L. An unrooted phylogenetic tree was constructed using HXK protein sequences from *A. thaliana* (AtHXK), *O. sativa* (OsHXK), *P. edulis* (PeHXK), *M. esculenta* (MeHXK), *B. napus* (BnHXK), *G. hirsutum* L. (GhHXK), *G. raimondii* (GrHXK), and *G. arboreum* (GaHXK), and are displayed in purple, green, yellow, red, light blue, dark blue, dark red, and orange, respectively.

According to the phylogenetic tree of HXKs from multiple species (Figure 4 and Supplementary Figure 4), the HXKs protein sequences were divided into four groups (Clade I, II, III, and IV). Two GhHXKs were classed into Clade I, four GhHXKs was classed into Clade II, 11 GhHXKs were grouped in Clade IV. However, no GhHXK were grouped in Clade III; only HXKs from monocotyledons *O. Sativa* and *P. edulis* were grouped into Clade III.

### Protein Features of GhHXKs

**FIGURE 5:**
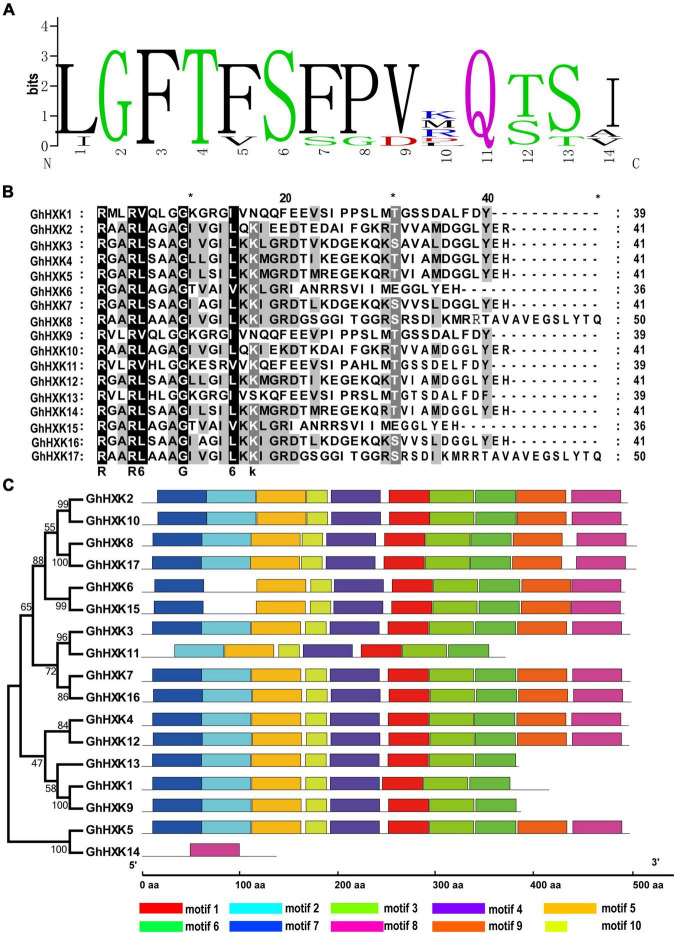
Protein features of HXKs in *G. hirsutum* L. **(A)** The weblogo of GhHXK protein glucose-binding sites. **(B)** The conserved adenosine binding sites of GhHXKs. **(C)** The motifs of HXK sequences from *G. hirsutum* L.

The protein sequence of GhHXKs was aligned using ClustalW software to characterize the protein structures. The amino acid sequence alignment showed 39–99% identity between GhHXKs members (Supplementary Figure 4), based on previous work analyzing HXK proteins in *A. thaliana*, *O. sativa, P. edulis*, *M. esculenta*, and *B. napus* (Cho et al., 2006; Geng et al., 2017; Zheng et al., 2020). The adenosine phosphate binding domain (Supplementary Table 3) and glucose-binding domain were found in most GhHXKs (Supplementary Figure 4 and Supplementary Table 4). The core glucose-binding domain of GhHXKs was conservative as “I/L-GFT-F/V-S-F/S-P/G-V/D” (Figure 5A). There is no glucose-binding domain in GhHXK14 (Supplementary Figure 4), while an intact adenosine phosphate binding domain exists in GhHXK14 (Figure 5B). The adenosine phosphate binding domain has a conserved motif of “RX_2_R-V/L-X_3_GX_3_-I/L/V” in GhHXKs, except for GhHXK9, GhHXK11, and GhHXK13 (Figure 5B). Sequences alignment showed that most of the GhHXKs are conservative with adenosine phosphate and glucose-binding domain.

Furthermore, the GhHXK protein motif characteristics were analyzed using the MEME online software, and ten conservative motifs were identified in the *GhHXK* gene family (Figure 5C). The majority of GhHXK proteins contain at least eight motifs, except for *GhHXK11* and *GhHXK14*, which have seven and one motifs, respectively.

### Duplication Analysis of *GhHXKs*

By searching the HXK domain against the genomes from chlorophyta to lycophytes plant species (Supplementary Figure 5), we found that *HXK* gene family members increased from low to high plant species. The chlorophyta species have less than two *HXKs*; however, lycophytes plants have more HXKs numbers than five.

**FIGURE 6:**
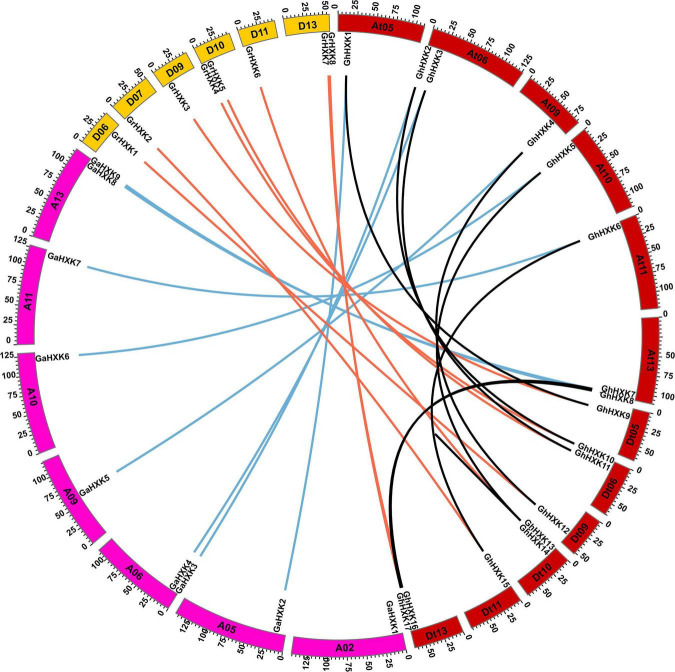
Circos plot showing *GhHXKs* paralogous gene pairs and orthologous gene pairs. Black lines connect the paralogous *GhHXKs* gene pairs. Red lines connect the paralogous gene pairs between *GhHXKs* and *GrHXKs*. Blue lines connect the paralogous gene pairs between *GhHXKs* and *GaHXKs*.

Therefore, to illustrate the duplication events of the *HXKs* gene on chromosome segments, the evolution of *GhHXK* genes was analyzed in *G. hirsutum* L., *G. arboreum* and *G. raimondii*, respectively, using MCScanX software. The results demonstrated that 16 *GhHXK* genes were derived from segmental duplication (accounting for 94.12%) of *GhHXK* gene family members, while only *GhHXK14* was derived from dispersed distribution on the chromosomes. Five *GaHXKs* were derived from segmental duplication, accounting for 55.56% of the total gene family members. Three *GaHXKs* were derived from dispersed distribution (accounting for 33.33%), and one *GaHXK* was a singleton gene. There are four *GrHXKs* derived from segmental duplication (accounting for 50%), three *GrHXKs* derived from tandem duplication events, and only one derived from dispersed distribution (Figure 6 and Supplementary Table 5). Duplication analysis demonstrated that segmental duplication is the leading cause of *HXK* genes duplication in cotton species.

Selection pressure refers to the evolutionary force of natural selection, which dictates the survival and reproduction of adaptive organisms. We further analyzed the Ka, Ks, and Ka/Ks ratios of the orthologous gene pairs in *G. hirsutum* L., paralogous gene pairs between *G. arboreum* and *G. hirsutum* L., and *G. raimondii* and *G. hirsutum* L. (Supplementary Table 6). The Ka/Ks ratios for the *GhHXKs* versus *GaHXKs* orthologous pairs ranged from 0.0784 to 0.819 and the Ks ranged from 0.00571 to 0.0510, suggesting that the orthologous pairs diverged 1.10 million years ago (MYA). The Ka/Ks ratios for the *GhHXKs* versus *GrHXKs* orthologous pairs ranged from zero to 1.917 and the Ks ranged from 0.0055 to 0.0317, suggesting that the orthologous pairs diverged from 1.06 MYA.

### *Cis*-Promoter Analysis of *GhHXKs*

**FIGURE 7:**
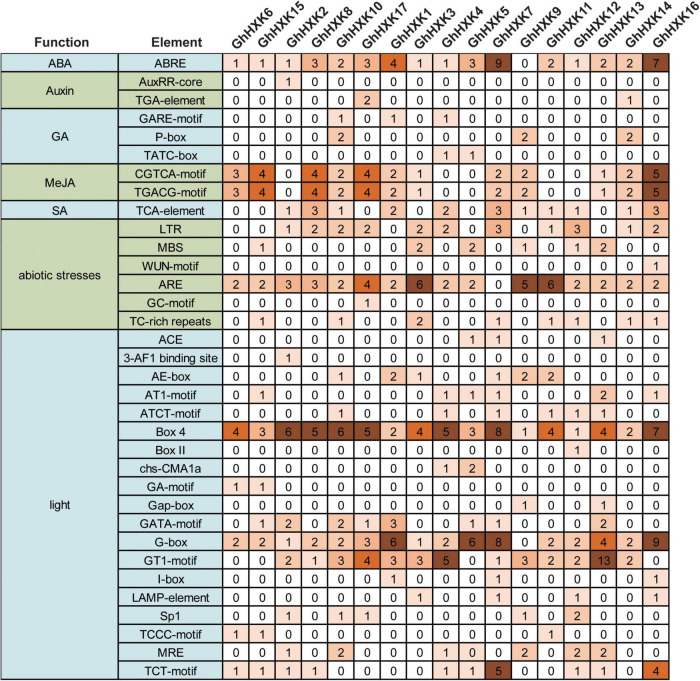
*Cis*-elements in the promoters of *GhHXK* genes. Numbers in the box are the number of *cis*-elements.

We further analyzed the *cis*-regulatory elements in the promoter regions of *GhHXKs*. The *cis*-acting elements that we identified in *GhHXKs* promoters were classified into three categories, including light-, hormone- and abiotic stress-responsive promoters (Figure 7). Light-responsive elements were identified in all *GhHXKs’* promoters. Of them, G-box was the most abundant (54) and was found in the promoters of 17 *GhHXKs*. Analysis of hormone-related elements demonstrated that the number of abscisic acids (ABA)-responsive elements were highest (43), followed by methyl jasmonate (MeJA)-responsive elements (32). Except for *GhHXK9*, all *GhHXK* promoters contain ABA-responsive elements (ABRE). *Cis*-acting elements involved in MeJA (TGACG-motif and CGTCA motif) were found in the promoters of 12 *GhHXKs*. The promoters of 11, 6, and 3 *GhHXKs* contain SA-, GA-, and Auxin-responsive elements, respectively. Additionally, all *GhHXK* promoters had at least two hormone-responsive elements, and *GhHXK14* contained all five hormone-responsive elements.

### *GhHXKs* Genes Differentially Expressed in Different Tissues and Fiber Developmental Stages

To illustrate the spatial expression patterns of *GhHXKs* genes, we analyzed the transcriptomes of various tissues (stamen, anther, seed, fiber, ovule, petal, calycle, torus, leaf, stem, root, cotyledon, stigma, and pistil) in G. *hirsutum* L. Transcripts of *GhHXKs* were detected in all tissues (Supplementary Figure 6), while their expressions exhibit a tissue-specific expression pattern in *G. hirsutum* L.

**FIGURE 8:**
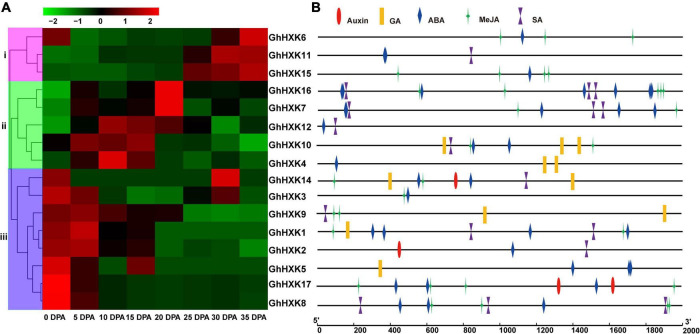
RNA-seq analysis of *GhHXKs* genes during fiber development at 0, 5, 10, 15, 20, 25, 30 and 35 DPA **(A)**, and plant hormone-related *cis*-elements in the *GhHXK* promoter regions **(B)**. The transcriptome data were normalized by fragments per kilobase of transcript per million mapped reads (FPKM) and visualized using the pheatmap software (https://www.omicshare.com/tools/Home/Soft/heatmap). The colorful bars from green to red indicate the expression levels from low to high, respectively.

**FIGURE 9:**
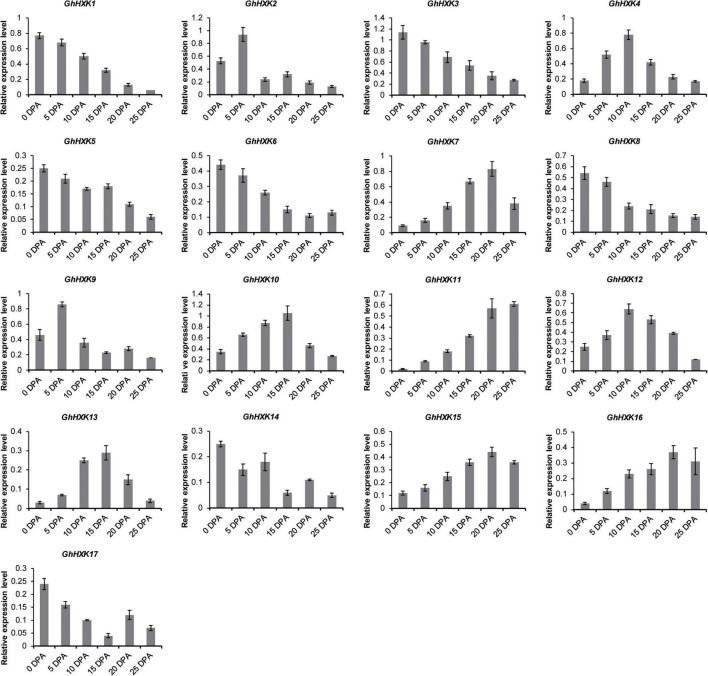
The expression levels of *GhHXKs* during fiber developmental stages (0, 5, 10, 15, 20, and 25 DPA) analyzed by qRT-PCR. Error bars represent means ± SE from three independent biological repetition. The relative expression level was calculated by using *GhUBQ7* as the internal control.

*Gossypium hirsutum* L. is one of the most important textile crops in the world. Considering its importance, we investigated the expression profiles of *GhHXK* genes during the fiber developmental stages at 0, 5, 10, 15, 20, 25, 30, and 35 DPA (Figure 8A). According to the expression patterns of *GhHXKs* during the fiber development process, *GhHXKs* expression patterns were classified into three groups: (i) secondary cell wall synthesis, where *GhHXK6*, *GhHXK7, GhHXK11*, *GhHXK15*, and *GhHXK16* were highly expressed 20–45 DPA, the; (ii) the elongation process during fiber development, where *GhHXK4*, *GhHXK7*, *GhHXK10*, *GhHXK12*, and *GhHXK16* had higher expression levels in fibers from 10 DPA to 20 DPA; and (iii) the fiber initiation and elongation process, where *GhHXK1*, *GhHXK2*, *GhHXK3*, *GhHXK5*, *GhHXK8*, *GhHXK9*, *GhHXK14*, and *GhHXK17* were highly expressed at 0 and 5 DPA. These transcriptome data were also verified by qRT-PCR experiments in Figure 9. *GhHXKs* have similar expression pattern both in qRT-PCR experiments and transcriptome data during fiber developmental stages.

**FIGURE 10:**
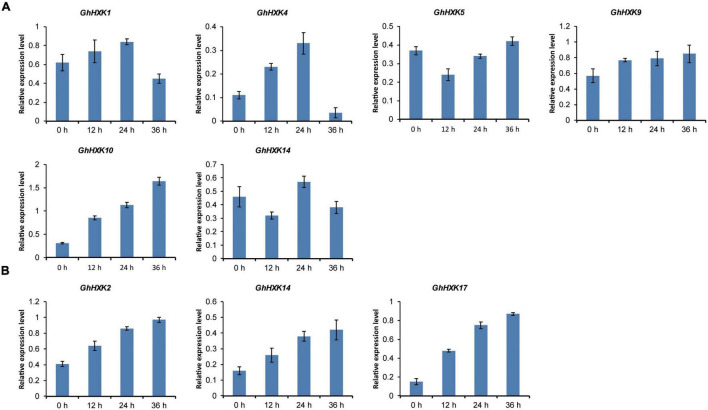
*GhHXKs* expression levels are induced by GA **(A)** and auxin **(B)** analyzed by qRT-PCR. GA treatment induced expression of most *GhHXKs* with GARE-motif, P-box or TATC-box in their promoter regions **(A)**. The relative expression levels of *GhHXKs* with AuxRR-core or TGA-element in their promoter regions **(B)**. The error bars represent means ± SE from three independent biological repetition. The *y*-axis represents the relative expression level. The *x*-axis represents the 0 DPA ovules treated with GA or IAA for 0, 12, 24, and 36 h, respectively.

The promoters of *GhHXK1*, *GhHXK4*, *GhHXK5*, *GhHXK9*, *GhHXK10*, and *GhHXK14* contain GA-responsive *cis*-elements, while the promoters of *GhHXK2*, *GhHXK14*, and *GhHXK17* have auxin-responsive *cis*-elements that are highly expressed from 5 to 20 DPA (Figure 8B). These genes can also be induced by GA and auxin treatment (Figure 10). Our results indicated that *GhHXKs* are involved in regulating the fiber development process and that the promoters of *GhHXKs* (i and ii) contain auxin- and GA-responsive elements.

## Discussion

Hexokinase (HXK) is an enzyme that catalyzes hexose phosphorylation during the metabolism of sugar, which functions as an energy substance and signal during plant growth. In this work, we identified 17, nine, and eight *HXKs* from *G. hirsutum* L., *G. arboreum*, and *G. raimondii*, respectively, and analyzed the *GhHXKs* chromosomal locations, phylogeny, gene structure, conservative motifs, duplicated types, *cis*-elements, and expression patterns during fiber development.

### GhHXKs Are Conservative Both in Nucleotide and Protein Sequence Levels

*Gossypium hirsutum* L. (2n = 4X = 52) is an allotetraploid cotton species. It originated approximately 1–2 MYA after the hybridization of two diploid cotton species, *G. arboreum* (2n = 2X = 26) and *G. raimondii* (2n = 2X = 26) (Galau and Wilkins, 1989). In this work, we identified 17, 9, and 8 *HXKs* from *G. hirsutum* L., *G. arboreum*, and *G. raimondii*, respectively. The total numbers of *GaHXK* and *GrHXK* equal that in *G. hirsutum* L.

Duplication events are the primary reason for the expansion of each gene family member. Analysis of the synteny and phylogeny of *HXKs* in the *G. hirsutum* L. genome demonstrated that *GhHXKs*, *GaHXKs*, and *GrHXKs* duplicated due to segmental duplication. The Ks and Ka were more significant in paralogous gene pairs (GhHXKs) than in orthologous gene pairs (*GhHXKs* vs. *GaHXKs* and *GhHXKs* vs. *GrHXKs*), and in the divergence time in paralogous gene pairs (*GhHXKs*) than between orthologous gene pairs. This indicates that duplication events in *GaHXKs*, *GrHXKs*, and *GhHXKs* occurred before the divergence of *G. raimondii* and *G. arboreum*. Additionally, the HXK sequences were conservative among cotton species.

Hexokinases in higher plants typically contain nine exons, such as *PeHXKs* (Zheng et al., 2020), *OsHXKs* (Cho et al., 2006), and *MeHXKs* (Geng et al., 2017). These nine exons were also found in most *GhHXKs*. Phylogenetic analysis of HXK proteins found more clade numbers among monocotyledons and fewer clade numbers among dicotyledons. The GhHXKs, GaHXKs, GrHXKs, AtHXKs, MeHXKs, and BnHXKs were clustered into three clades (I, II, and IV), while OsHXKs, PeHXKs were clustered into four clades (I, II, III, and IV). This indicates that the *HXKs* of monocotyledonous plants had a higher mutation level than that of dicotyledons.

### The Central Hypothesis Role of GhHXKs in Sugar Signal Transduction During Fiber Development

Sucrose is the primary photosynthesis produce and is transported to growing cells, such as fiber cells. Sucrose is a disaccharide made up of glucose and fructose, and functions as an osmotic substance and raw material for the synthesis of cell wall cellulose. Sucrose synthase (SuSy) and invertase are involved in the first step of sucrose degradation by cleaving the glycosidic bond between glucose and fructose (Cabello et al., 2014). SuSy helps break down sucrose into fructose and UDP-glucose for cellulose biosynthesis. A SuSy protein, SusC, is highly expressed during the synthesis of the secondary cell wall in fibers and the cell wall fraction. The subcellular location of the protein demonstrated that SusC is localized on the cell wall, which could indicate the presence of UDP-glucose function in cellulose and callose synthesis (Brill et al., 2011). Jiang et al. (2012) demonstrated that over-expressing *GhsusA1*, a cotton *SuSy* gene, increased the thickness of the secondary cell wall and overall fiber strength, which indicates that a sucrose signal is involved in controlling cellulose biosynthesis in the development of cotton fiber (Jiang et al., 2012). When this synthetic *SuS*y gene is overexpressed in cotton, the transgenic cotton plants showed longer fiber length, enhanced fiber strength, and increased cellulose contents (Ahmed et al., 2020). The cell wall invertase (CWIN) is responsible for sucrose cleaving into fructose and glucose, while the expression levels of *GhCWIN* are significantly more highly expressed at 5 and 10 DPA than 15 and 20 DPA (Wang and Ruan, 2012). During the fiber development process, the sucrose content decreased from fiber initiation (0 DPA) to fiber elongation (12 DPA), and was accompanied by increasing in glucose and fructose in fiber content (Sun et al., 2019). Both SuSy and CWIN can catalyze sucrose into monosaccharides and contribute to cotton fiber development by providing component hexoses for cellulose synthesis. Additionally, the production of hexoses can increase the content of osmotic substances content, which contributes to turgor pressure for fiber elongation (Weschke et al., 2003).

The synthesis and elongation of cotton fiber cell could be required to obtain higher energy. Studies have found significantly higher ATP synthase activity in 10 DPA wild-type fiber cells than in ovule samples and leaf samples. Additionally, exogenously applying the inhibitors of ATP synthase, piceatannol (PA), and oligomycin (OM) decreased fiber length and lowered the ATP/ADP ratio (Pang et al., 2010). Other studies demonstrated that phosphorylated glucose participated in the pentose phosphate pathway, which provides NADPH for cellular respiration (Lu et al., 2016), and that HXKs catalyzes the irreversible step of glycolysis, which provides energy for cell growth (Aguilera-Alvarado and Sanchez-Nieto, 2017).

Transcriptome analysis demonstrated that the hexokinase inhibitor NAG, which repressed cotton fiber elongation, depends on the glucose signal transduced by HXKs (Li et al., 2021). Glucose phosphorylation is also involved in the synthesis of inositol, a signal molecule (Loewus et al., 1982), which positively regulates cotton fiber length. The inositol synthase enzyme, *myo*-inositol-1-phosphate synthase, positively regulates fiber elongation. The *GhMIPS1D* gene was ectopically expressed in the *Arabidopsis mips1* mutant showed longer root cells and a higher plant height (Ma et al., 2019). RNAi MdMIPS1/2 in apple promoted programmed cell death and necrosis, while apple necrosis was directly caused by the excessive accumulation of reactive oxygen species. Therefore, apple necrosis could be associated with salicylic acid, which increased the polysaccharide-mediated cell wall (Hu et al., 2020).

### Crosstalk About IAA, GA, Glucose, and GhHXKs in Fiber Development

Gibberellin and auxin are two plant hormones that promote fiber elongation. Analysis of promoter *cis*-elements and expression data of expression profile and qRT-PCR experiments demonstrated that some *GhHXKs* are regulated by GA and IAA.

Gibberellin (GA) plays two roles when regulating the content of intracellular glucose. GA_3_ treatment can promote the accumulation of sugar in potato tubers under low-temperature conditions by inducing changes in the expression of genes involved in sugar accumulation, ADP-glucose pyrophosphorylase (AGPase) (Xie et al., 2018). In the presence of glucose, the GA synthesis enzyme, *GA20ox1*, can significantly up-regulated by KNO_3_ (Ikeya et al., 2020).

In the daytime, cytochrome C (*Cyt C*)-deficient *Arabidopsis* accumulates glucose with lower levels of GA, while GA treatment complements this reduction of glucose accumulation in Cyt C-deficient plants (Racca et al., 2018). GA synthesis was suppressed by glucose, and the application of mevalonic acid could break down this suppression. Therefore, the key enzyme of the isoprenoid pathway was the target of C-catabolite suppression (Bruckner, 1992). GA_3_ repressed the transcriptional levels of *HXK1* and *HXK2*, which negatively interfered with the transduction of glucose signals, depending on hexokinase phosphorylation in grape berries (Zhang et al., 2014).

Abscisic acids negatively regulates cotton fiber development, and other studies demonstrated that ABA crosstalked with glucose signal transduction. During the germination process of rice seeds, high glucose concentrations delayed seed germination by repressing ABA catabolism (Zhu et al., 2009). The enzyme UGT73C14 utilized UDP-glucose as sugar donors for ABA glycosylation in *G. hirsutum* L., and the UDP-glucose can be synthesized by UTP and phosphorylated glucose (Glc-Pi) (Gilbert et al., 2013).

The glucose sensor HXK mutant *gin 2* is also resistant to exogenous auxin (Moore et al., 2003). Glucose affects most of the genes regulated by auxin metabolism (Mishra et al., 2009). High glucose concentrations reduced the root meristem zone by repressing the auxin transporters, *PIN1* accumulation, and reducing auxin levels in Arabidopsis roots (Yuan et al., 2014). Most IAA-regulated genes were transcriptionally regulated by glucose alone; however, glucose antagonistically functions on IAA-regulated genes (Gupta et al., 2009).

**FIGURE 11:**
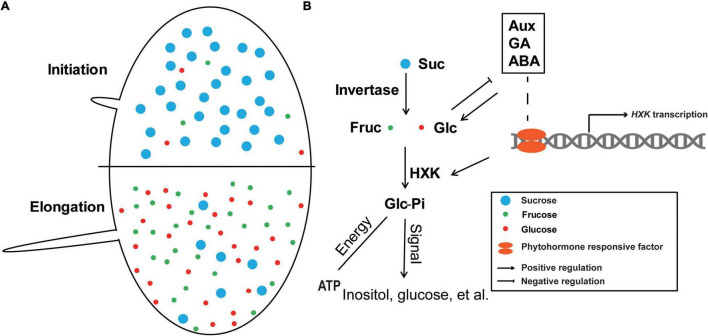
A hypothesis about the roles of GhHXKs in cotton fiber development. **(A)** During fiber developmental stages, from initiation to elongation, the sucrose content decreased, and the fructose and glucose content increased. **(B)** The phosphorylated hexose functions in energy supply and signal transduction in fiber development; Meanwhile, intracellular hexose levels are regulated by IAA, GA, and ABA, and *GhHXK* is the center of this pathway. The blue, red and green dots represent sucrose, glucose and fructose, respectively. The arrows and blunt arrows indicate positive and negative regulation of the specific processes, respectively.

Above all, glucose functions as a molecular signal that crosstalks with IAA, GA, and ABA, while HXK-catalyzed glucose-phosphate is the core of glucose signal transduction. Analysis of the promoter *cis*-elements analysis and RNA-seq data demonstrated that *GhHXKs* contain GA- and IAA-related *cis*-elements can also be regulated by these phytohormones. This indicates that various hormones can crosstalk HXKs with sugar signals when regulating the development of cotton fiber (Figure 11).

## Conclusion

We performed a genome-wide characterization of the *GhHXK* gene family in cotton research by identifying chromosomal distribution, gene structure, phylogenetic analysis, duplication events, promoter *cis*-elements, and spatial-temporal expression of the *GhHXKs*, which provides a comprehensive analysis of the *GhHXK* gene family.

## Data Availability Statement

The datasets presented in this study can be found in online repositories. The names of the repository/repositories and accession number(s) can be found in the article/Supplementary Material.

## Author Contributions

GX and XZ: conceptualization. LD: writing – reviewing. ZL and HL: software and methodology. HW: perform experiments, revise, and writing sections of the manuscript. All authors contributed to the article and approved the submitted version.

## Conflict of Interest

The authors declare that the research was conducted in the absence of any commercial or financial relationships that could be construed as a potential conflict of interest. The reviewer JG declared a shared affiliation with the author XZ to the handling editor at the time of review.

## Publisher’s Note

All claims expressed in this article are solely those of the authors and do not necessarily represent those of their affiliated organizations, or those of the publisher, the editors and the reviewers. Any product that may be evaluated in this article, or claim that may be made by its manufacturer, is not guaranteed or endorsed by the publisher.

## Footnotes

1
http://www.cottongen.org

2
https://www.arabidopsis.org/

3
https://www.ncbi.nlm.nih.gov/nuccore

4
http://brassicadb.cn/#/

5
https://www.ncbi.nlm.nih.gov/genome

6
http://smart.embl.de/

7
https://www.ncbi.nlm.nih.gov/cdd

8
http://gsds.gao-lab.org/

9
https://meme-suite.org/meme/tools/meme

10
https://web.expasy.org/protparam/

11
https://wolfpsort.hgc.jp/

12
http://sphinx.rug.ac.be:8080/PlantCARE

13
https://www.omicshare.com/tools/Home/Soft/heatmap

## Supplementary Material

Supplementary Figure 1 Chromosomal distribution of *GaHXKs* and *GrHXKs*. The arrows show the direction of the transcription of *HXK* genes.

Supplementary Figure 2 Chromosomal distribution of *GanHXKs, GstHXKs, GloHXKs*, and *GroHXKs*. The arrows show the transcript direction of *HXK* genes.

Supplementary Figure 3 Phylogenetic relationships and gene structures of HXKs in cotton species. **(A)** Phylogenetic tree of HXKs from *G. hirsutum* L., *G. arboreum*, *G. raimondii*, *G. anomalum*, *G. stocksii*, *G. longicalyx*, and *G. rotundifolium*. The tree was constructed by MEGA 7.0 with default parameters with 1,000 bootstraps. **(B)** Gene structure. The blue oval and black lines represent exons and introns, respectively.

Supplementary Figure 4 Amino acid alignment analysis of GhHXKs. The red lines indicate the regions with different functions. The red arrow directs the eight conserved amino acids (counting the amino acid residues based on GhHXK2, the eight amino acids are Asp-106, Thr-110, Lys-196, Asp-231, Gly-253, Asp-438, Gly-440, and Ser-473), which are the active residues of hexokinases (HXKs). The alignment was performed with ClustalW and displayed by GeneDoc.

Supplementary Figure 5 Distribution of the HXK family genes in plantae. The phylogenetic tree of 18 plant species was shown on the left **(A)**. The genome size **(B)** and number of *HXK* genes **(C)** found in each genome are indicated on the right.

Supplementary Figure 6 Expression heatmap of the *GhHXKs* at various tissues (stamen, anther, seed, fiber, ovule, petal, calycle, torus, leaf, stem, root, cotyledon, stigma, and pistil).
