# Supplementary figures and images for: The hexokinase Gene Family in Cotton: Genome-Wide Characterization and Bioinformatics Analysis
Source: Front Plant Sci. 2022 May 16;13:882587. doi: 10.3389/fpls.2022.882587 (PMC9149573; doi:10.3389/fpls.2022.882587)

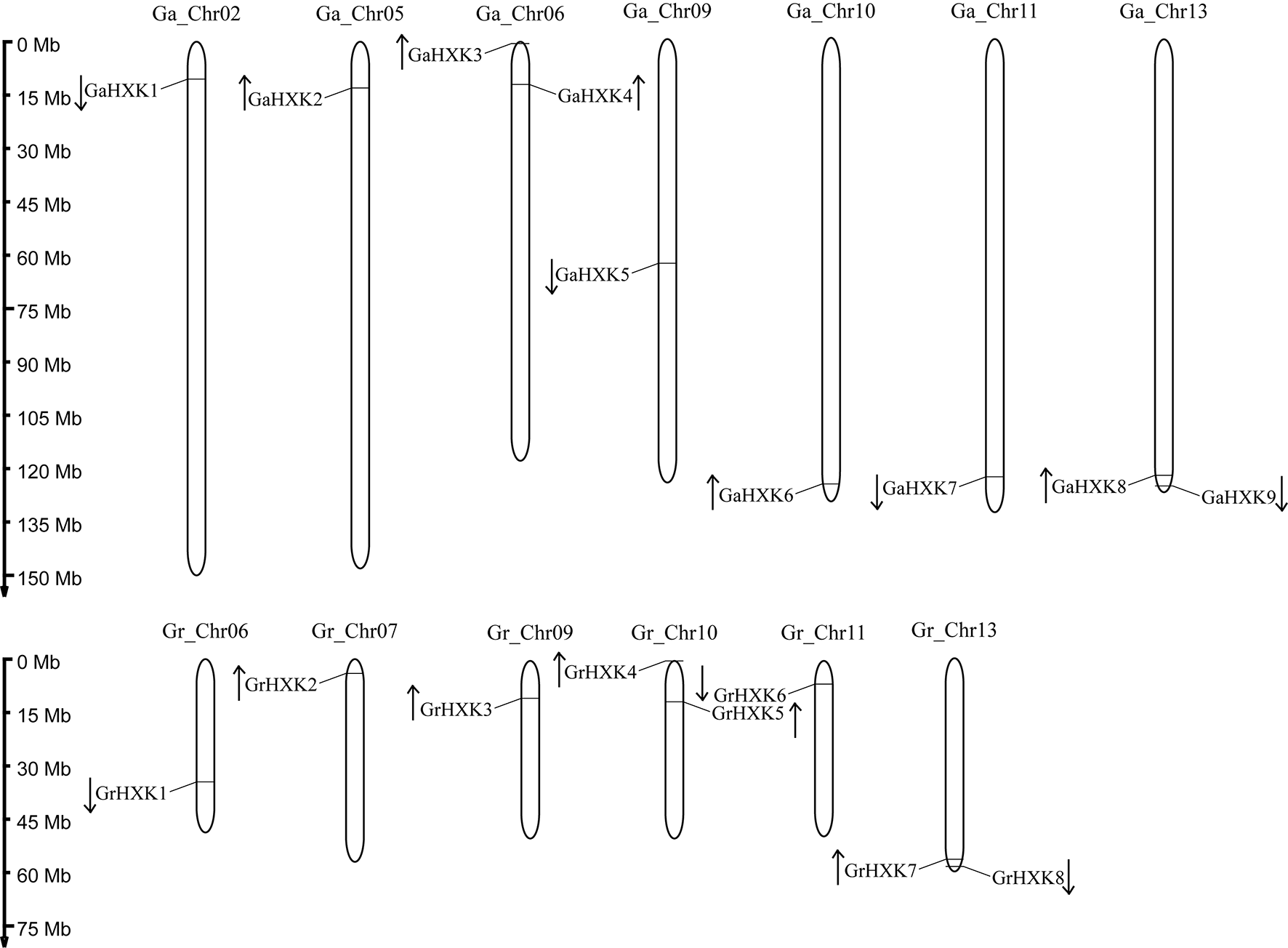

Supplement: Supplementary Figure 1 — Chromosomal distribution of GaHXKs and GrHXKs. The arrows show the direction of the transcription of HXK genes. [file Image_1.TIF]

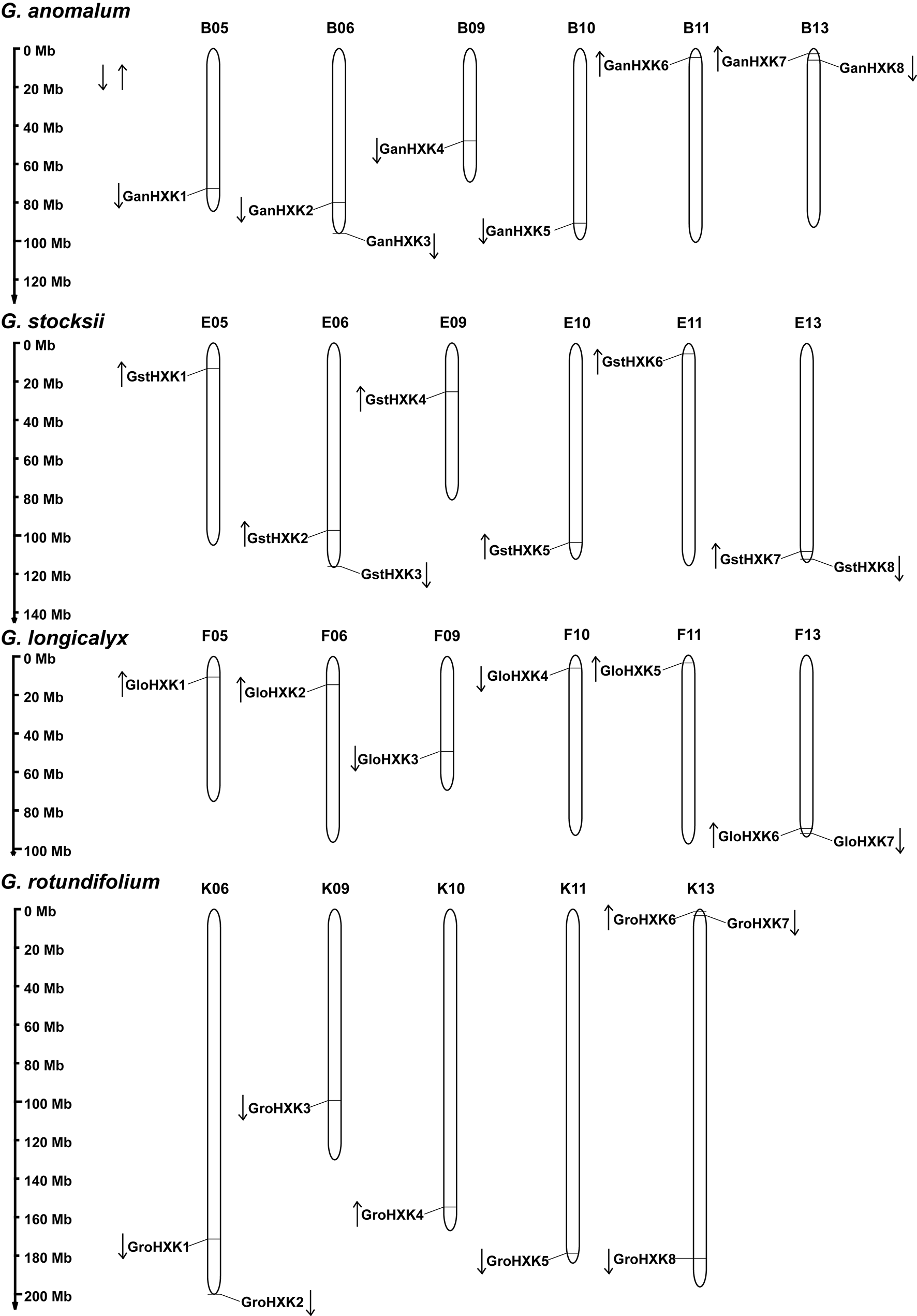

Supplement: Supplementary Figure 2 — Chromosomal distribution of GanHXKs, GstHXKs, GloHXKs, and GroHXKs. The arrows show the transcript direction of HXK genes. [file Image_2.TIF]

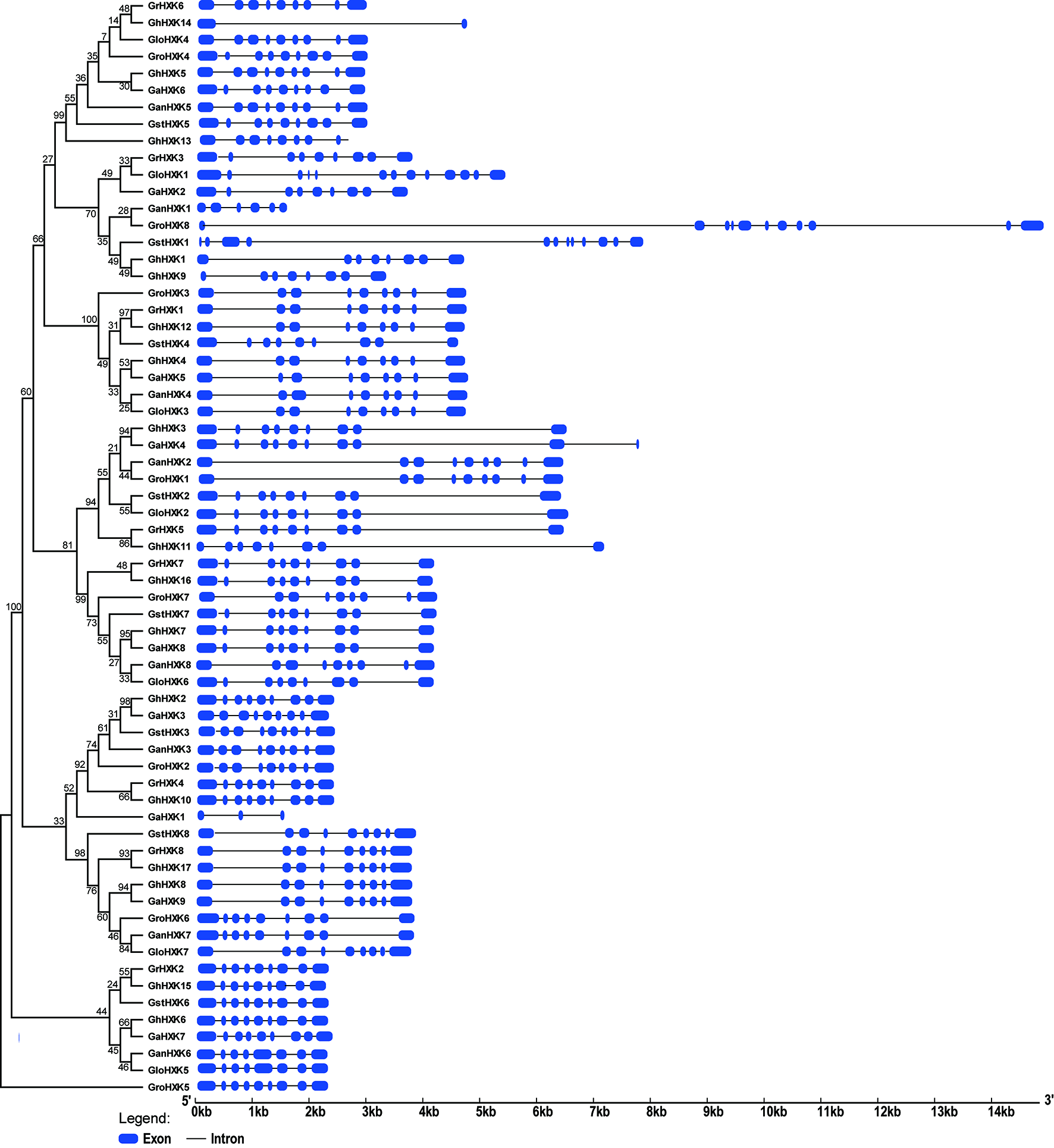

Supplement: Supplementary Figure 3 — Phylogenetic relationships and gene structures of HXKs in cotton species. (A) Phylogenetic tree of HXKs from G. hirsutum L., G. arboreum, G. raimondii, G. anomalum, G. stocksii, G. longicalyx, and G. rotundifolium. The tree was constructed by MEGA 7.0 with default parameters with 1,000 bootstraps. (B) Gene structure. The blue oval and black lines represent exons and introns, respectively. [file Image_3.TIF]

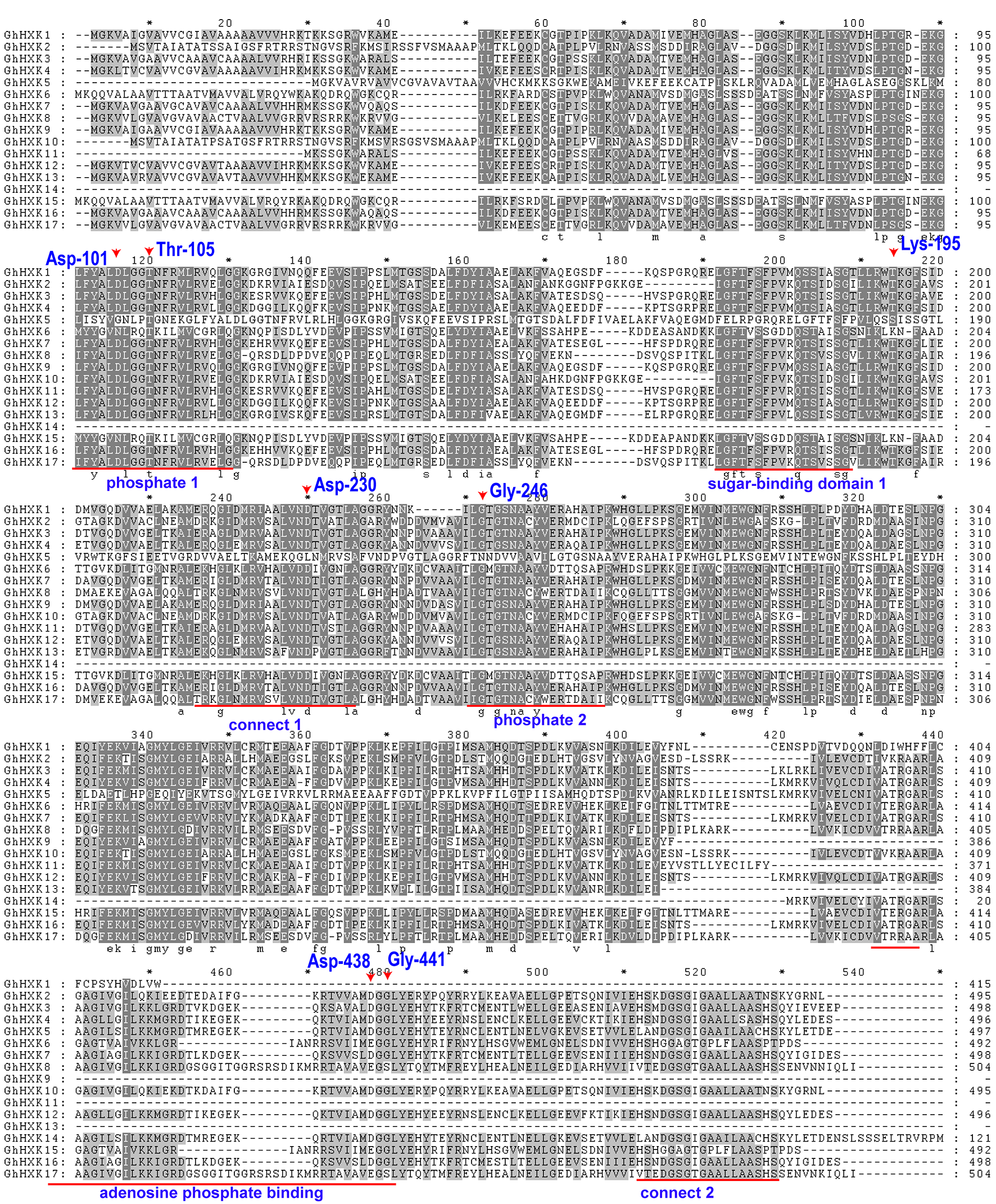

Supplement: Supplementary Figure 4 — Amino acid alignment analysis of GhHXKs. The red lines indicate the regions with different functions. The red arrow directs the eight conserved amino acids (counting the amino acid residues based on GhHXK2, the eight amino acids are Asp-106, Thr-110, Lys-196, Asp-231, Gly-253, Asp-438, Gly-440, and Ser-473), which are the active residues of hexokinases (HXKs). The alignment was performed with ClustalW and displayed by GeneDoc. [file Image_4.TIF]

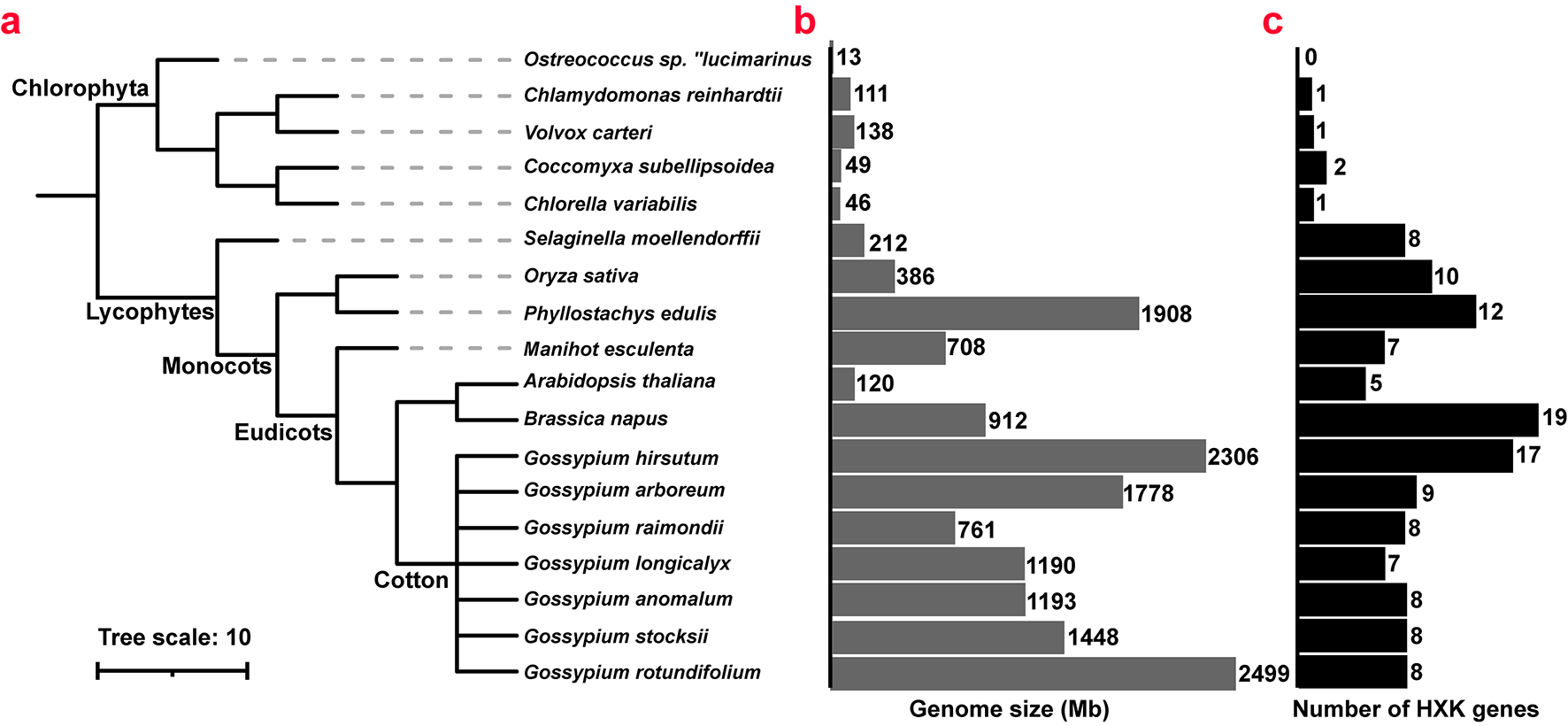

Supplement: Supplementary Figure 5 — Distribution of the HXK family genes in plantae. The phylogenetic tree of 18 plant species was shown on the left (A). The genome size (B) and number of HXK genes (C) found in each genome are indicated on the right. [file Image_5.TIF]

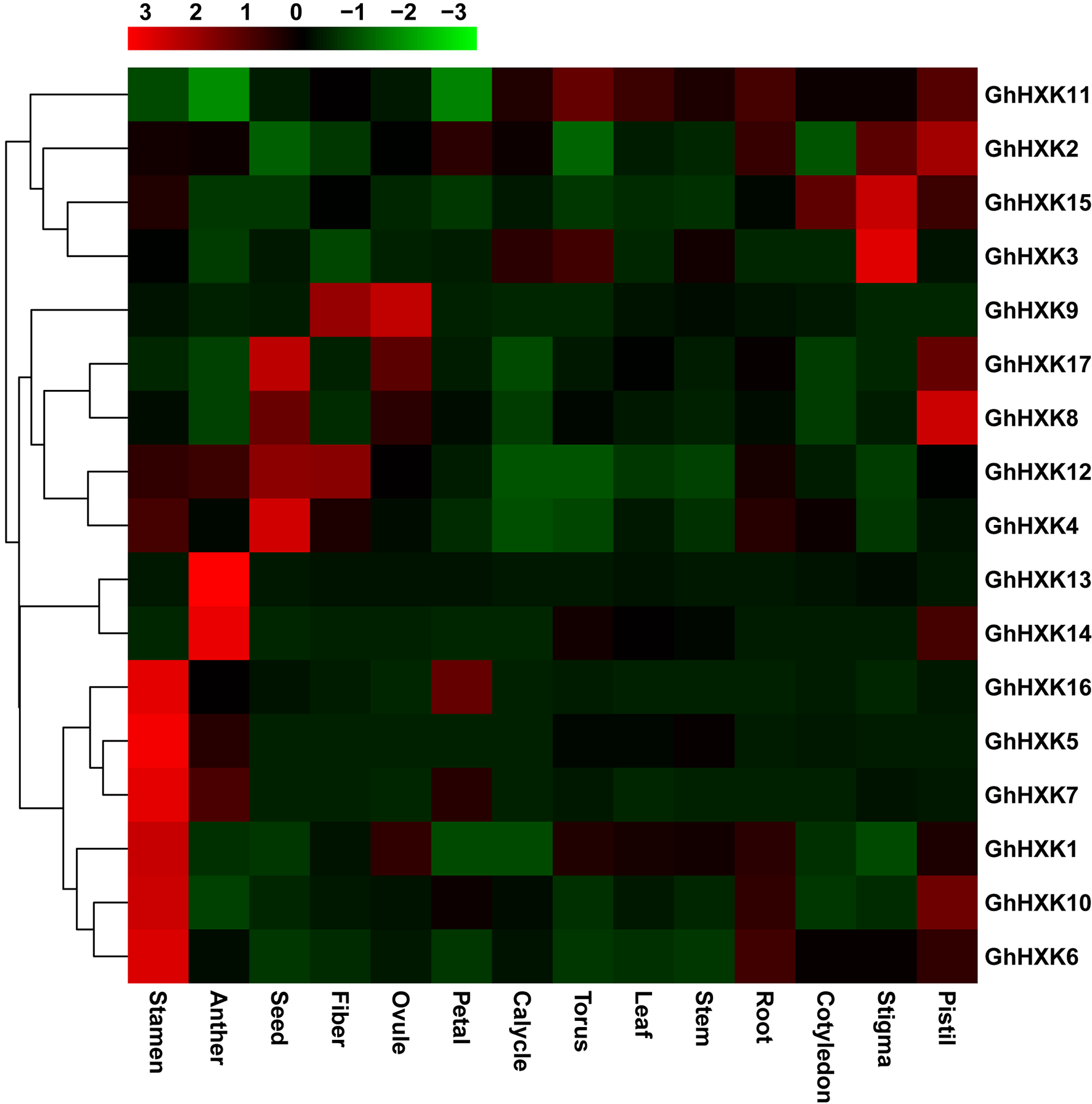

Supplement: Supplementary Figure 6 — Expression heatmap of the GhHXKs at various tissues (stamen, anther, seed, fiber, ovule, petal, calycle, torus, leaf, stem, root, cotyledon, stigma, and pistil). [file Image_6.TIF]
